# A Treatise on the Nervous Diseases of Women

**Published:** 1841-07-01

**Authors:** 


### A TREATISE ON THE NERVOUS DISEASES OF WOMEN:—COMPRISING AN INQUIRY INTO THE NATURE, CAUSES, AND TREATMENT OF SPINAL AND HYSTERICAL DISORDERS. By *Thomas Laycock*, M.D. &c. &c. London, 1840.

THE diseases of females have always held a prominent place in medical literature. For whether we regard the protean form they often assume—their frequent danger—or the interesting subjects of them ; they possess peculiar claims on our solicitude and attention. In another point of view the study of these diseases is most important; for there is no class of maladies the treatment of which imposes greater responsibility on the medical attendant; or where his character and skill are more frequently placed in jeopardy.

Hence we took up the volume before us with considerable interest and pleasure: and the perusal of the sensible preface led us to hope that new views, or new lights would be thrown on the pathology and treatment of the class of diseases which the author professes to have carefully studied, and respecting which he comes forward to instruct his readers. It is difficult to pass a judgment in limine on works of this kind. Authors and readers have different tastes and often different objects in view. Hence a good book is not unfrequently censured because it does not chime in with the readers' preconceived notions, or meet the exigencies, or motives, for which he reads it. For instance, the work before us will be perused with far different feelings by the naturalist or speculative theorist, than by the medical man who reads for practical information.

Dr. Laycock seems to be aware of this, and endeavours to obviate the objections that may be made to speculative and theoretical medical publications.

"I have often thought," he states in his preface, "that treatises on the practice of medicine professing to be free from theory and to contain nothing more than a description of diseases, and the methods of treatment, are of questionable utility. The condensed style in which they are usually written admits of no detailed exposition of the principles laid down, or of the facts from which those principles are deduced. The writer consequently appears to dictate rather than to state an argument; to be the occupant of a professorial chair, rather than a fireside companion. The interest which physiology might give to the subject is lost sight of; and thus the work is dry and uninteresting, and never studied."

We do not accord with this opinion of our author. On the contrary, we think that the practical works which he thus describes and condemns, are not only the most studied, but deserve to be so.

Theory may assist us in generalizing recorded facts, but in our opinion few discoveries in medical science have resulted from it; and it is often a fallacious guide in the practical treatment of disease. "Ars medica tota in observationibus," is an adage we believe not more trite than true.

Works therefore containing a faithful description of the symptoms, diagnosis, and prognosis of diseases, with the treatment which experience has proved to be the most efficacious, are those to which we constantly refer; and which enable us to decide in the hour of difficulty, when the mere theorist is bewildered in his own mazes. So far from "never being studied," these publications are by far the most popular: we need only, to exemplify our meaning, refer to that of "Pemberton on the Abdominal Viscera." This work, although it contains no theory, is enlivened by no physiological views, and does not evince more than ordinary research, is yet in every body's hands, and why?—because it enables us, by its graphic description of diseases, to identify them—to discriminate them from others—to foretell their probable termination—and to decide at once on their treatment. It is in vain that we look for these desiderata in Dr. Laycock's book. Although it is a sort of monograph, being confined to one class of diseases; yet we may turn over the whole of the pages before we find the connected symptoms of any one of those diseases, or the treatment specifically laid down. We have the "disjecta membra"—detached thoughts—theories—anomalies—and symptoms of disease, and the treatment of those *symptoms*:—but we want a more lucid arrangement, an identification of disease—a design, and an end.

The defective and scattered arrangement of the work also renders it difficult to read, and to ascertain the author's meaning; and this difficulty is increased by the references to authorities being made at the bottom of the page by letters, instead of by the author's names. We are thus compelled to turn back generally three or four times in a page to a long list of authors in the commencement of the book, to know from whom he quotes, or whose opinion he controverts.

Notwithstanding these remarks, we have found in Dr. Laycock's book much to admire; it displays considerable research and contains some original and novel views: yet, we are compelled to repeat, that it neither comes up to the standard of what we think such a work should do, nor will it, in our opinion, fulfil the object which the author himself seems to have had in view.

He has collected a great many facts and circumstances in natural history—such as the periodic changes in insects; the nidification of birds; the period of gestation in animals; instances and proofs that male birds and animals have different tints and colours from the female; that the latter at certain times emit peculiar odours; that emasculation reduces the male, and removal of the ovaries the female, to an intermediate state of colour, &c. &c., with a variety of other similar observations.

Dr. Laycock is of opinion, that many of these facts throw light on the changes and diseases of the female system, and that without a knowledge of them many anomalies would be inexplicable. We do not think that he is borne out in these assertions by the use he makes of the facts, in their application to explain either the causes of disease, or the treatment. Many of his theories are fanciful, others mere assumptions; they may be probable or true—but they add but little to our previous knowledge of the subject. We are far from wishing to disparage the study of natural history in reference to medical science. On the contrary, we think a knowledge of the habits, instincts, and diseases of animals, and of their comparative anatomy, is essential to the study of the diseases of mankind. Our acquaintance with many facts in human physiology and pathology is derived entirely from this source; but we tread on slippery ground when we deduce theories from it for the explanation of the causes of human disease.

We shall, however, better explain our meaning as we proceed in our analysis of this volume.

Dr. Laycock commences the special physiology of hysteria by assuming or establishing four general principles.

1st. The nervous system is the seat of hysteric disease.

2ndly. Hysteria is peculiar to females.

3rdly. Women of susceptible nervous system are more liable than others; and,

4thly. Hysteric diseases appear only during that period of life in which the reproductive organs perform their functions.

We have but little to object to these principles so far as they go: indeed they are generally admitted.

Dr. Laycock, however, controverts the opinion which is commonly held, that the uterus is the exciting cause of that disturbance of the nervous system which occasions the phenomena of hysteria.

Dr. Bright states, that "the peculiar condition of the nerves in hysteria seems to owe its origin, more or less directly to the extensive nervous sympathies of the uterus, which are capable of being anatomically demonstrated as well as pathologically inferred." Dr. Copland and others advocate very similar doctrines. "It will be observed, however," says our author, "after a careful perusal of these various theories, that they afford no satisfactory explanation of many of the peculiar phenomena of hysteria; of the spasm of the glottis; of the remarkable embonpoint of many hysterical patients under the most meagre diet; of the occasional profuse salivation; of the periodicity observed in the paroxysmal forms; of the more frequent occurrence of the disease in Spring and Autumn; or indeed of a hundred other circumstances connected with its multiform varieties." p. 7.

Now we think that a theory may be a very good one, which explains the usual phenomena of a disease, although it may not account for the anomalies which occasionally attend it. These anomalies may arise from other concurrent causes. With the exception of the first, the symptoms above enumerated are all anomalous: at any rate they are not "peculiar" or essential to the malady. In attempting to explain the various phenomena of disease we are apt to be too exclusive, and because we cannot account for many deviations which may arise during its progress we abandon our theory, forgetting that, in the complex machine with which we have to do, many disordered and inexplicable actions may be set up, although the exciting cause may be essentially the same. Theory is only really useful when it leads to successful treatment, and for this reason we believe that the theory which refers the symptoms of hysteria to uterine disturbance has been productive of the greatest advantage. It is true many vague hypotheses have been framed, to explain *how* the uterus acts to produce hysteria, and in what manner medicines operate to remove it; but the facts and practices on which they were founded still remain: for nature and the laws of the human body are yet the same. We may not be able to account for all the phenomena which may occasionally occur in so complex a disease as hysteria, for analogous symptoms may arise from opposite causes—or, vice versa, opposite conditions of the uterine organs may occasion similar symptoms; but in the main, our treatment will be successful in proportion as we are acquainted with the morbid sympathies of the uterine system, and direct our curative measures accordingly. In the work before us we find many sensible suggestions for the removal of certain symptoms, but the general treatment of hysteria is not very clearly or systematically laid down, nor is the disease itself distinctly or graphically described.

Dr. Laycock's object, indeed, does not appear to have been to give a methodical description of female disorders, or a systematic mode of treatment, but rather to explain them and their anomalies, by analogous reasoning from facts collected from natural history and other sources. We do not think, as we before remarked, that the inferences he deduces from many of these facts are so valuable as he seems to regard them. They do not much advance our previous knowledge of the diseases of the female system, nor lead to any striking novelty in the way of treatment. We shall, therefore, confine our few remaining remarks to a brief consideration of some of those points of the work to which he invites the especial attention of his readers.

Dr. Laycock states in his preface that, "In the second part of the work the comparison between the diseases of infantile dentition and of puberty, the demonstration and illustrations of the connexion between the gouty diathesis and diseases of the nervous system, the chapter on the pathology of the passions, the relations of paroxysmal diseases to other affections and to the encephalon, and the different endowments of the lateral halves and extremities of the nervous centres, will, I humbly think, offer points of interest both to the pathologist and practitioner."

He commences the first inquiry by shewing that, previously to puberty, the brain is in a condition different from that which is observed after puberty. This, he thinks, "is evident from the impulse given to the intellect on the accession of the latter; and the difference is still more obvious when we consider the effects of cerebral injuries before and after adolescence. While these are among the most fatal injuries which can happen to the adult, in children their consequences are not more grave than those which would follow a lesson (lesion) of corresponding severity in any other part of the body." "Since the cerebrum and generative organs are both undeveloped, we can look for no symptoms specially involving these; but excluding these two sources of excitement and of anomalous symptoms, we have," in the first dentition, "all the remaining phenomena of hysteria. Those usually mentioned by systematic writers may be enumerated as follows :—1. Those originating in organs connected with the upper extremity of the spinal cord; *a.* symptoms originating in the cranial cord—coma, watching, and sudden starting from sleep, increased sensibility of the surface, general and epileptic convulsions, opisthotonos, paralysis; *b.* symptoms implicating the face—strabismus, fixed stare, sardonic grin, trismus, convulsive twitchings; *c.* symptoms referred to the larynx, trachea, and bronchi—aphonia, convulsive cough, hydrophobic gasp, spasmodic closure of the glottis, croupy breathing, wheezing from increased or diminished secretion from the bronchi; *d.* pharynx, œsophagus, and stomach—retching, vomiting, dysphagia, eructation, impaired appetite; *e.* heart—palpitation, syncope; *f.* respiratory muscles—dyspnœa, sneezing, hiccup, yawning. 2. Symptoms implicating the dorsolumbar portion of the cord—paralysis and tetanic extension and flexion of the lower extremities, serous exudation from the buttocks, increased micturition, ischuria, mucous discharges from the urethra, dysuria, constipation, diarrhœa, colic, tympanitic distention. Systematic writers in general agree in making one or other of these symptoms the cause of the rest, the less grave having the blame of originating the graver; so that the protrusion of a tooth through the gums, constipated bowels, or flatus, have had the whole catalogue attributed to their injurious influence. That they are exciting causes may be readily granted; but if dentition necessarily caused such serious symptoms, why are they not observed in every infant? or at the second evolution of the teeth? or when the dentes sapientiæ appear? It is true that a constipated state of the bowels will excite convulsions in infants: but why more readily in infants than in adults? during the cutting of a tooth than in the interval? or why, indeed, is there constipation at all?" Why indeed!

We have made this long extract to shew the style and mode of reasoning of our author. He huddles together a catalogue of symptoms, which, he says, are "usually mentioned by systematic writers," as characteristic of one disease—which would apply to all the diseases of the nervous system; but, so far from the above symptoms being usually observed in hysteria or teething, the life of a practitioner may be spent without witnessing a third of them in either. He then assumes that these systematic writers agree to make one or other of these symptoms the cause of the rest. We confess we are unacquainted with such writers. We do not, however, with Dr. Laycock, call the protrusion of a tooth through the gums *a symptom*—but the cause of the symptoms, simply because, in nine cases out of ten, the division of the gum will remove them. Again, he states that, because the brain, previously to puberty, can bear external injury with impunity, it is inirritable or "insensible;" because convulsions, &c., occur both in teething and hysteria, they are analogous affections. We apprehend that they who have had the most experience in infantile diseases, will pause before they admit with our author, that the brain is not excitable or prone to disease during childhood. On the contrary, almost all who have attended to the subject will agree, that the brain of children is especially obnoxious to disease from even slight causes of irritation.

This increased susceptibility of morbid action probably arises, as it is generally supposed, from its large supply of blood and consequent early development.

It will be readily granted that convulsions occasionally occur, both in hysteria and in the teething of children—but to render a parallel or analogy between these two diseases really useful, they must be characterised by similar causes, symptoms, prognoses, pathological conditions, and modes of treatment; examined by this test, we humbly think the connexion between dentition and hysteria will offer few points of interest either to the pathologist or to the practitioner.

Dr. Laycock next traces an analogy between hysteria and gout. He states that they arise from a similar cause—have the same pathological connexion—and that the same remedies are applicable to both.

This is his theory.—"In an individual of gouty diathesis an excess of urea, or other urinary constituent, being produced, and, at the same time, in consequence of impaired action of the kidneys a diminished amount being eliminated, the excrementitious matter (like digitalis and other poison) accumulates in the system, and at last overcharging it, causes an 'explosion' (to use the word of Boerhaave) to take place, in the form of a gouty paroxysm, or failing this, excites irritation of different organs in succession, and produces in men the different forms of erratic gout,—in women, of anomalous hysteric disease; the symptoms varying in each individual, as one or other organ weaker than the rest falls under its noxious influence, so that in one case they will be those of mania, in a second of thoracic disease, in another of epilepsy, in another of the hysteric paroxysm, or assume the form of angina pectoris, spasmodic asthma, or local neuralgiæ." 170.

That the pale urine of hysteric females contains but little urea is well known, but we hesitate before we admit the inference, that because but little is eliminated, there exists an excess in the system, and that this excess is the materies morbi. Dr. Laycock's theory appears to us to be little better than mere assumption. Friendly as we are to the humoral pathology, we must have further proofs than he brings forward before we admit that the presence of urea, or any other urinary constituent, in the system, is the cause of some of the most grave diseases to which mankind is liable. With regard to gout we entirely coincide, however, in the views Dr. Holland and Dr. Prout have brought forward, and we think that their researches will tend to a rational mode of treatment. The former eminent physician thinks, that the existence of some peccant matter in the system, probably in the blood, characterizes the gouty diathesis, and that its elimination constitutes the gouty paroxysm.

Dr. Prout's opinion, in the recent edition of his valuable work, is thus stated:—"According, therefore, to these views, the lactic and lithic acids, considered with reference to rheumatism and gout, may be regarded somewhat in the light of *materies morborum*, or, strictly speaking, the undue presence of these acids in the urine, or elsewhere, under certain circumstances, may be viewed as indices of the existence of certain diseased actions going on in the primary tissues of the body, and which are known by the names of rheumatism and gout." *

These views thus cautiously expressed are clear and definite, and point out the rationale of the preventive and curative measures of gout and rheumatism, but they have but little connexion with hysteria, in which, if we mistake not, an opposite condition of the system exists.

They would lead to an erroneous treatment of this latter disease, and can serve no useful purpose in explaining either the symptoms, causes, or pathological condition.

Dr. Laycock refers to Dr. Conolly and Dr. Ferriar to substantiate his theory of the close analogy between gout and hysteria. We accordingly turned to these valuable writers, and although we do not think Dr. Laycock is happy in these references, as regards his own views, yet the passages are so well expressed, and put the resemblance between hysteria and gout in so proper and practical a point of view, that we take the liberty to quote them.

Dr. Conolly says :—" Some of the older writers laid considerable stress on the influence of a gouty constitution in predisposing to hysteria. Facts of this kind are not easily verified, and hysteria may occur in a gouty family without being really connected with a gouty constitution. There can be no difficulty in an age when a new and more enlightened pathology of the fluids seems to be dawning, in admitting so much of the ancient humoral pathology as to allow that either a gouty, or any other morbific matter in the blood, may be the occasional exciting cause of those nervous irritations which characterize a susceptible temperament, just as in other cases. The same morbific matter, by irritating the nerves of the extremities, appears to excite the common pains of gout and rheumatism. The nervous irritation in these latter examples is sufficiently well established: the existence of a morbific matter yet remains to be proved." †

* Stomach and Urinary Diseases, by Dr. Prout ; p. 211.

† Cyclop. of Pract. Med., art. Hysteria ; p. 571.

The passage to which Dr. Laycock refers in Dr. Ferriar is the following. "There is a strong resemblance between hysteria and gout in the power of counterfeiting different diseases, *but with this material distinction,* that the hysterical representations are commonly void of danger, while those produced by gout are often more dangerous than the simple disorders which they imitate. The hysterical hæmoptöe, for example, is seldom productive of bad consequences—but the arthritic apoplexy, pneumonia and cardialgia, are much more alarming, and run their course quicker, than similar complaints originating from other causes. But these diseases agree in this respect, that the accession of the regular paroxysm puts a favourable period to the irregular symptoms of each."*

In the chapters "on the pathology of the passions—the relations of paroxysmal diseases to other affections of the encephalon—and the different endowments of the lateral halves and extremities of the nervous centres"—many novel and hypothetical views are advanced which display considerable research and ingenuity. But as they do not elicit much practical information, we shall pass them over, and select some of our author's remarks on the treatment of disease, which we think will be more interesting to our readers.

In this, however, the therapeutic part of the work, we have the same fault to find as in the descriptive; we have no definite plan of treatment laid down, or rules given by which the precise adaptation of remedies may be made. The divisions and subdivisions of hysteria are innumerable. We have hysteric catarrh, hysteric bronchitis, hysteric tetanus, hysteric hepatitis, hysteric constipation, &c.

We very much question whether this method of applying the epithet "hysteric" to distinct diseases simplifies our knowledge of them: on the contrary, we think it tends to confuse and embarrass. It is well known that the peculiar feature or characteristic of hysteria is to simulate other diseases, as has been ably shewn by Dr. M. Hall, Sir B. Brodie, Ferriar, and others; and great skill on the part of the practitioner is often required to discriminate the feigned from the real disease. But to designate the latter hysteria simply because it occurs in a constitution prone to hysteria, is, we think, improper—because it tends to divert our attention from its real nature, as well as from its appropriate remedies.

In the general treatment of hysteria Dr. Laycock strongly recommends air and exercise, and those means which tend to divert and exhilarate the mind, and give tone and strength to the body. In that form of hysteria which is occasionally observed in habits hereditarily predisposed to gout, he gives the following formula, which is also a great favorite with him in some other anomalous hysterical affections.

"℞. Ferri sulph. gr. xv.
Pulv. colocynthidis gr. ij.
Pil. hydrargyri gr. xij.
Extract. colchici acet. gr. ix.
Extract. gentian. gr. xxx. M. et fiat pilulæ xij.
One to be taken three times a day."

---

* Med. Histories and Reflections, Vol. 2, p. 43.

One of the predisposing causes of hysteria is a neglected and confined state of the bowels: and a train of ill health in females may often be traced solely to this source. We were not however aware of the extent to which this neglect is carried, until we met with this startling passage.

"*Constipation.*—It is surprising how long a period delicate women will occasionally allow to elapse between each evacuation of the bowels; from five to ten days is by no means an unusual time, and sometimes as many weeks have intervened. The constipation of hysterical females depends partly upon paralysis of the intestine, and partly upon diminished secretion from the mucous surfaces: unless there be a local cause it is always proportionate to the severity of the general disease, and accompanied by more or less ischuria, tympanitis, abdominal and spinal tenderness, &c.

It is in the severe cases of hysteria that the most extraordinary constipation has been witnessed; in such, from three to six months, and even seven years, have elapsed without a stool." 244.

In many of the gastric affections of females Dr. Laycock speaks highly of the nitrate of silver, and the trisnitrate of bismuth. We bear our willing testimony to the efficacy of the latter remedy in gastrodynia and pyrosis, which are common diseases in females. It is usually given in doses of from five to ten grains, with an equal quantity of magnesia twice or thrice daily, and when the pain is severe, with the addition of half a grain of opium. In protracted cases the nitrate of silver is a valuable remedy, and was first introduced into notice by Autenrieth, Ruff, and Dr. James Johnson. Dr. Laycock gives the following formula from Dr. Steinitz of Greiffenberg.

"℞. Argenti nitr. crystal. gr. v.
Solve in aquæ distillatæ, q. s. et adde
Extracti taraxaci
Pulv. rad. glycyrrh. āā q. s. ut fiat massa in pilulas xx. dividenda.

One or two of these pills to be taken in the morning and again in the evening, with mucilaginous drinks."

The sections on spinal tenderness and distortions will be read with interest. They contain extracts from Stromeyer and other writers on these subjects, and point out a correct diagnosis and treatment of these affections.

On the whole, the work of Dr. Laycock evinces considerable extent of reading and industry of research: and although it has been our misfortune to differ from him in our estimate of the value of some of his opinions and theories, as well as of the general arrangement of his book, yet we take leave of him with a high opinion of his talents, and of his zeal for the improvement of his profession.

